# Renal Subcapsular Hematoma Post Transradial Catheterization

**DOI:** 10.7759/cureus.77536

**Published:** 2025-01-16

**Authors:** Venkata Gandi, Dwayne Mohan, Monica Kovuri, Brandon Rockwell, Edward Distler

**Affiliations:** 1 Internal Medicine, South Georgia Medical Center (SGMC) Health, Valdosta, USA; 2 Cardiology, South Georgia Medical Center (SGMC) Health, Valdosta, USA

**Keywords:** critical care, renal subcapsular hematoma, retroperitoneal hemorrhage, subcapsular hemorrhage, transradial catheterization

## Abstract

In this case report, we describe the first documented renal subcapsular hematoma following transradial cardiac catheterization. Traditionally, this occurs via a transfemoral approach for cardiac catheterization. Initially presenting with chest pain, the patient underwent a successful percutaneous coronary intervention (PCI) via radial approach. This procedure involved the placement of a drug-eluting stent in the right coronary artery via transradial access. The patient received over 8000 units of heparin during percutaneous coronary intervention to achieve an activated clotting time (ACT) of 242 seconds. However, post intervention, the patient's condition became critical, revealing a right renal subcapsular hematoma as the underlying cause of his hemodynamic instability.

## Introduction

Over a million cardiac catheterizations in the United States of America are performed annually to diagnose coronary artery disease (CAD), arrhythmias, valvular heart diseases, heart failure, and congenital heart anomalies [1]. Since 1989, the transradial approach for percutaneous coronary intervention (PCI) and coronary angiography has gained popularity due to its significant benefits [2]. These include a decrease in mortality rates, major bleeding incidents, and complications at the access site and shorter hospital stays [3]. Interestingly, about 80% of hematomas and retroperitoneal bleeding cases post heart catheterization are effectively managed with supportive care, including IV fluids and blood transfusions [4]. To date, there are no cases of retroperitoneal hematoma in transradial access for PCI. In most cases, retroperitoneal hematomas happen spontaneously [5]. It Is crucial to explore and document these rare complications.

## Case presentation

**Figure 1:**
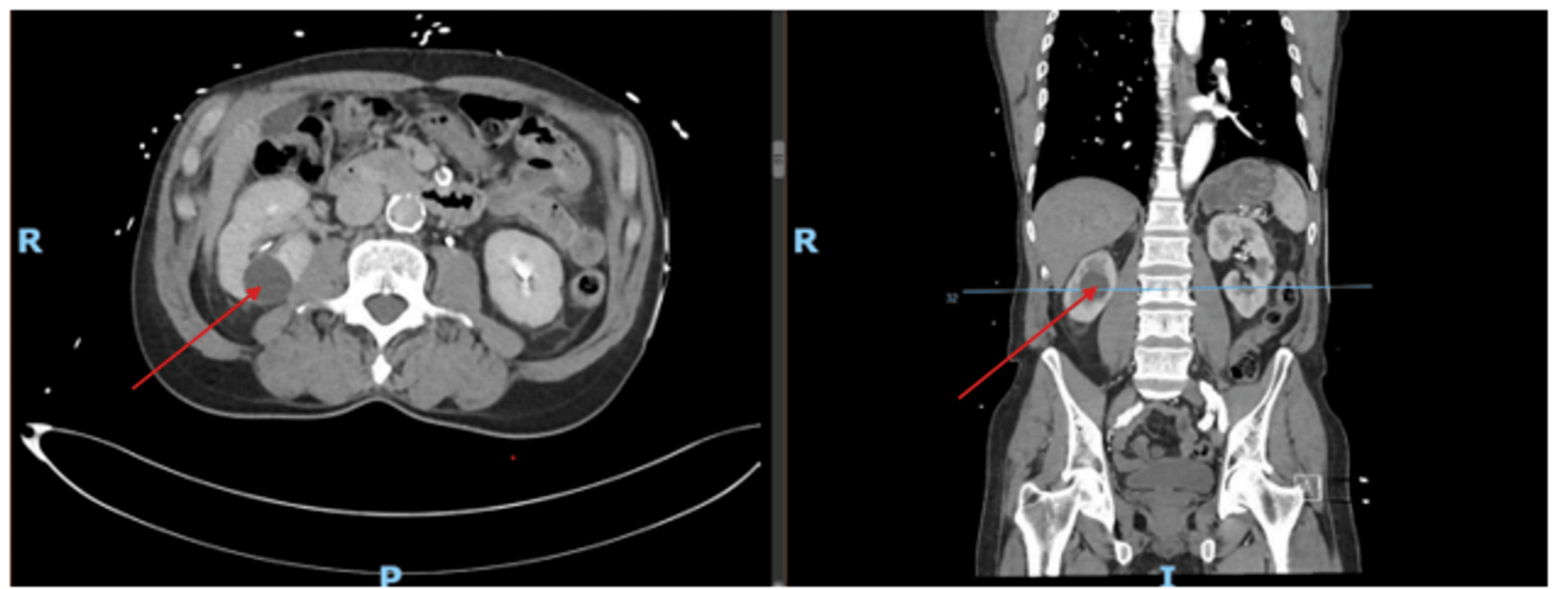
Axial and sagittal views of simple cysts shown bilaterally on day 0 of the patient's arrival at the hospital (see red arrows, which point to a cyst on the right kidney). There is no retroperitoneal hemorrhage or renal subcapsular hematoma present.

A 66-year-old man, with a history of essential hypertension, a history of nonobstructive coronary artery disease, type 2 diabetes mellitus, hyperlipidemia, and a significant smoking history, presented to the emergency department with substernal chest pain. Despite being hemodynamically stable and electrocardiogram displaying no ST segment changes, his chest pain persisted. The patient's high-sensitivity troponin trended up to 793.5 ng/L (normal: <19.8 ng/L). An initial CT scan revealed bilateral renal cysts and mild atherosclerotic changes but no dissection (Figure 1).

The patient underwent a transradial percutaneous coronary intervention with drug-eluting stent placement in the right coronary artery after loading heparin to the therapeutic range. However, within an hour post procedure, he developed abdominal pain, bradycardia, and hypotension.

**Figure 2:**
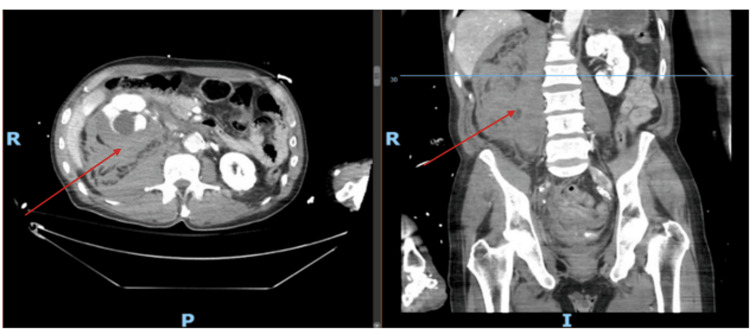
Repeat CT scan (axial image on the left and sagittal image on the right) after the patient had abdominal pain after heart catheterization. Renal subcapsular hematoma measuring 10×9×7 cm is shown. Retroperitoneal hemorrhage is present too (see red arrows). Axial and sagittal views are shown.

Subsequent imaging revealed a significant new finding: a large subcapsular hematoma on the right kidney, measuring 10×9×7 cm, causing mass effect, and an extensive retroperitoneal hematoma (Figure 2). These hematomas were not present in earlier scans, indicating that they were acute developments post cardiac catheterization.

The patient's condition was complicated by acute blood loss anemia, as indicated by the drop in hemoglobin levels from 15.3 g/dL to 7.4 g/dL. Our patient required a total of five units of packed red blood cells during his hospitalization at our facility. The patient underwent endoscopic evaluation for gastrointestinal bleeding. However, no gastrointestinal bleeding was found. It was determined that the patient was most likely experiencing perinephric hemorrhage due to a rupture of renal cyst present on imaging prior to percutaneous coronary intervention.

Conservative therapy with IV fluids and blood transfusion is generally the treatment for renal subcapsular and retroperitoneal hematoma [6]. When medical management fails to improve a patient's hemodynamic status, patients with this condition should undergo angiography with arterial embolization performed by interventional radiology (IR) [5]. If IR is not available, the hematoma will need to be evacuated, and the bleeding artery will have to be ligated [5].

Our patient received IV fluids and blood transfusions. However, due to his hemodynamic instability, he was transferred to another facility. The patient underwent arterial embolization by interventional radiology and was transferred back to our facility once he was hemodynamically stable. The patient was subsequently discharged home from our facility. The patient followed up with his cardiologist in one month, and he was able to resume his daily activities with ease. He continued aspirin and clopidogrel as his hemoglobin remained stable. He had no further episodes of hemodynamic instability due to acute blood loss after his arterial embolization at the outside facility. This case highlights a rare but serious complication of the transradial approach in percutaneous coronary interventions.

## Discussion

Transradial access for percutaneous coronary intervention (PCI) is preferred over transfemoral access due to its lower risk of vascular and bleeding complications. However, this method is not without its challenges, including potential complications such as radial artery perforation, spasm, dissection, forearm hematoma, compartment syndrome, occlusion, pseudoaneurysm, arteriovenous fistula, nerve damage, and infection [7]. Notably, factors such as anticoagulation, antiplatelet therapy, certain medical conditions, and lifestyle choices such as smoking can elevate the risk of complications, including the rare occurrence of renal subcapsular hematoma [8]. Spontaneous subcapsular renal hematoma may occur due to the possibility of an undetectable renal tumor in patients in the absence of anticoagulation, arteritis, or trauma [9].

In our case, a male patient with a history of smoking, hypertension, and type 2 diabetes mellitus underwent PCI via the transradial approach. Smoking, known to accelerate atherosclerosis, increases the risk of plaque dislodgement during heart catheterization [8]. Additionally, the presence of bilateral renal cysts in our patient heightened the risk for renal complications [10]. Post procedure, a CT scan revealed a significant right renal subcapsular hematoma, measuring 10×9×7 cm, a novel complication following radial access.

While the transradial approach is generally safer, this case illustrates that complications such as renal subcapsular hematoma can still occur. Previous reports have primarily been associated with the transfemoral approach for cardiac catheterization. For instance, a 2007 study highlighted a case where plaque dislodgement at the aorto-renal junction led to renal hematoma following femoral artery catheterization [11]. Similarly, a 2014 report discussed renal hematoma post transfemoral angiography due to accidental damage to the renal artery [12].

We propose that the administration of heparin during coronary intervention significantly contributed to the development of renal subcapsular hematoma in our patient. Current practice guidelines suggest administering an initial heparin bolus dose, calculated based on patient weight, within the range of 70-100 units per kilogram. This dosage is aimed at achieving the desired activated clotting time (ACT) of between 250 and 300 seconds [13]. During the procedure, the patient was administered two boluses of heparin, each consisting of 4000 units to achieve a target-activated clotting time of 242 seconds. This substantial dosage of heparin, received during percutaneous coronary intervention, likely played a pivotal role in the onset of the subcapsular renal hematoma. Similarly, in 2016, a case of a rupture of a renal cyst of a 66-year-old man after receiving heparin bolus was reported [10]. The authors suspected that the rupture of the cyst was due to receiving heparin bolus [10]. Renal cysts have thin walls that are prone to rupture, leading to subcapsular renal hematomas. It is rare for a simple cyst to rupture [14]. We suggest that this risk for subcapsular renal hematomas is further exacerbated by anticoagulant use when a patient has known renal cysts, which impedes normal clotting processes, resulting in more extensive bleeding and hematoma formation, as observed in our patient.

Our case is pivotal in illustrating that while transradial access for PCI significantly reduces the risk of certain complications, it does not eliminate the possibility of rare occurrences such as renal subcapsular hematoma, especially in patients with preexisting risk factors and patients receiving high boluses of heparin during cardiac catheterization. This highlights the need for vigilant monitoring and a comprehensive understanding of individual patient risks during and after transradial cardiac catheterizations.

## Conclusions

The transradial approach in percutaneous coronary interventions (PCI) is widely regarded as safer compared to the transfemoral approach. This case is the first documented instance of renal subcapsular hematoma associated with the transradial approach in percutaneous coronary interventions (PCI). The presence of renal cysts significantly increases the risk due to their fragile walls, particularly with high bolus doses of heparin given during percutaneous coronary intervention. In addition, smoking, diabetes, and uncontrolled hypertension further elevate this risk for renal subcapsular hematoma and retroperitoneal bleeding. Management typically involves supportive care, but arterial embolization should be considered for ongoing hemodynamic instability. This illustrates the importance of careful anticoagulant management to enhance patient safety during PCI.
